# A Perspective on Ovarian Cancer Biomarkers: Past, Present and Yet-To-Come

**DOI:** 10.3390/diagnostics7010014

**Published:** 2017-03-08

**Authors:** Frederick R. Ueland

**Affiliations:** Department of Obstetrics and Gynecology, Division of Gynecologic Oncology and the Markey Cancer Center, University of Kentucky College of Medicine, Lexington, KY 40515, USA; fuela0@uky.edu; Tel.: +1-859-257-1613.

**Keywords:** biomarkers, ovarian tumor biomarkers, ultrasound, serial ultrasound, ovarian cancer

## Abstract

The history of biomarkers and ultrasonography dates back over more than 50 years. The present status of biomarkers used in the context of ovarian cancer is addressed. Attention is given to new interpretations of the etiology of ovarian cancer. Cancer antigen 125 (CA125) and multivariate index assays (Ova1, Risk of Ovarian Malignancy Algorithm, Overa) are biomarker-driven considerations that are presented. Integration of biomarkers into ovarian cancer diagnostics and screening are presented in conjunction with ultrasound. Consideration is given to the serial application of both biomarkers and ultrasound, as well as morphology-based indices. Attempts are made to foresee how individualized molecular signatures may be able to both provide an alert of the potential for ovarian cancer and to provide molecular treatments tailored to a personalized genetic signature. In the future, an annual pelvic ultrasound and a comprehensive serum biomarker screening/diagnostic panel may replace the much maligned bimanual examination as part of the annual gynecologic examination. Taken together, it is likely that a new medical specialty for screening and early diagnostics will emerge for physicians and epidemiologists, a field of study that is independent of patient gender, organ, or the subspecialties of today.

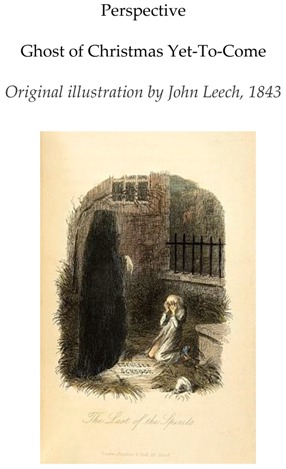

As one year closes and another begins, I find myself reflecting on ovarian cancer diagnostics. It is truly humbling how little we have accomplished in this field over the last half-century. All the while, the rest of the world has been busy. Since the first biomarker was reported, we have harnessed the atom and ushered in the Nuclear Age. Since the first *ovarian* biomarker was reported, we have invented the integrated circuit and spawned the dynamic Information Age. Yet, as gynecologic oncologists, we continue to struggle with the early identification of ovarian cancer and whether ovarian cancer actually begins in the ovary at all. The first serum biomarker for epithelial ovarian cancer was introduced in 1965 (carcinoembyonic antigen, CEA) [1]. This was a milestone in cancer diagnostics, as prior to this, oncologists were equipped with little to detect or monitor ovarian cancer. Keep in mind that this was when ultrasound was just emerging as a very rudimentary medical diagnostic instrument, and well before the advent of computed tomography (CT) or magnetic resonance imaging (MRI). Now some fifty years later, it is easy to ask, “Why haven’t we done more?” Perhaps the recent focus on molecular-genetic technology and personalized cancer treatment will inspire a new Diagnostic Age in oncology. I am an optimist at heart, and am hopeful that our biomarker story will read somewhat like the Charles Dickens novella, A Christmas Carol, where the return of Jacob Marley’s ghost 7 years after his death helps give clarity to the past, present, and yet-to-come.

First, it is important to clarify our diagnostic objective. My generation has believed, quite sensibly I think, that epithelial ovarian cancer arises from the ovary. Ovarian cancer has always utilized a taxonomy-based classification system first introduced in the 1930s, then validated by the World Health Organization’s Classification in 1973, and propagated into modern day. The story was as follows: ovarian epithelial inclusion cysts are trapped beneath the surface epithelium of the ovary and eventually undergo malignant transformation giving rise to invasive cancer. It was all a little mysterious and the association with ovulation was difficult to validate, but “incessant ovulation” did appear to be a significant risk factor. Until recently, true fallopian tube cancers were very rare. The historic requirement for the diagnosis of a fallopian tube cancer included the following: (1) the main tumor is grossly in the fallopian tube; (2) microscopically, the mucosa is chiefly involved and has a papillary pattern; and (3) if the tubal wall is involved to a great extent, the transition between benign and malignant tubal epithelium should be demonstrated [2]. Truthfully, many serous “ovarian cancers” probably do begin elsewhere and metastasize to the ovary since ovarian stromal involvement is the principle requirement to categorize a malignancy as primary ovarian cancer. Since serous peritoneal, fallopian tube, and ovarian cancers are histologically and morphologically similar regardless of where they begin, and are treated alike, they have been collectively categorized as ovarian cancer. Today, our approach to treatment is based on this premise, specifically that all these cancers are lumped together as one. National collaborative group trials for ovarian cancer have typically studied all three malignancies together rather than individually, even non-serous cell types. And this was very sensible, since we thought of ovarian cancer in terms of its anatomic origin and combining made practical sense for clinical trial accrual. This dilemma is apropos given the current belief that the fallopian tube (serous tubal intraepithelial carcinoma, STIC) may be the primary culprit in the etiology of many serous cancers of the ovary [3]. It is very helpful to know what the target is, not just for purposes of tidiness and taxonomy, but also for understanding how to envision the next generation of diagnostic tests.

Kurman and coauthors recently described the need for a paradigm shift in our understanding of ovarian cancer [4]. Endometrial precursors are likely responsible for many of the Type I ovarian cancers as endometrioid and clear cell types originate ostensibly from endometriotic implants. These are typically indolent, low-grade malignancies, and endometrioid, transitional and clear cell cancers with distinct molecular markers: KRAS, BRAF, ERB-2, PTEN and others, but not TP-53. And most gastrointestinal-type tumors involving the ovary are also secondary malignancies, with primary mucinous ovarian cancers comprising only 3% of all epithelial ovarian cancers. Fallopian tube precursors are likely the cause of the more common Type II, high-grade serous ovarian cancers which are characterized by TP-53 mutations. In the end, stromal and germ cell tumors may be the only true anatomic ovarian malignancies. The challenge of course, is that all gynecologic cancers are not organ-specific, so our diagnostic and treatment strategies need to evolve.

## 1. Past

The biomarker past was an era of single-marker diagnostics. CEA was first described in 1965 as a serum biomarker for mucinous colon cancer, and in 1976 as a blood test for women with ovarian cancer [1,5]. At the time, this was a tremendous advance in science. Not long after, cancer antigen 125 (CA125) was announced as a serum biomarker specific for ovarian cancer [6] (Table 1). To move from an age of very limited imaging and diagnostics to an ovarian cancer blood test was transformational. In retrospect, it can be argued that CA125 has done little to improve ovarian cancer care. The Food and Drug Administration (FDA) never approved CA125 for preoperative use in the United States, but only for cancer surveillance for women with a known diagnosis of ovarian cancer. Ironically, the majority of CA125 tests ordered today are for the evaluation of an ovarian tumor prior to surgery. The use of serum CA125 has also never been associated with a survival benefit, whether utilized before or after diagnosis. This may be an indictment of the test itself, of the disease, the stage at diagnosis, treatment options, or a combination of these factors.

Although CA125 is the best-known serum ovarian cancer biomarker, it is not the only one: CEA (mucinous), LDH (dysgerminoma, mixed germ cell tumors), β-hCG (choriocarcinoma, mixed germ cell tumors), inhibin B (granulosa cell tumors), α-fetoprotein (yolk sac tumors, embryonal cell tumors), and HE4 are also available. In 2008, HE4 was cleared by the FDA for use in monitoring patients with a known diagnosis of ovarian cancer, able to detect recurrence of epithelial cancers 2 to 3 months in advance of CA125. Like CA125, it does not have a preoperative diagnostic indication from the FDA. CA125 is the most studied biomarker for serous epithelial cancer arising from the ovary, fallopian tube, or peritoneal cavity, but it is neither a sensitive nor particularly specific cancer marker. This may partly explain why its use has not translated into an improvement in patient survival. For 35 years, we have been trying to overcome this biomarker’s inadequacy by combining it with other markers, combining it with imaging, or monitoring its behavior over time: all ultimately without epic success. Success, our patients have discovered, is identifying ovarian cancer in the earliest of stages where treatment can have a lasting impact on survival. Our understanding of protein biomarkers has improved recently as a result of advances in proteomic diagnostic technologies.

## 2. Present

In 2009, the FDA cleared the first preoperative serum biomarker test for ovarian cancer. After five years of diagnostic discovery and systematic clinical testing, a 5-protein biomarker panel named Ova1^®^ became the first multivariate index assay (MIA) to gain clearance in the United States [7,8]. Ova1 combines the second generation CA125-II with other inflammatory and transport proteins (transferrin, β-2 microglobulin, apolipoprotein A-1, and transthyretin) into a test result of low or high risk for ovarian cancer. The following year, a two-protein test was FDA-cleared that combined CA125 and HE4 (Risk of Ovarian Malignancy Algorithm, ROMA^®^) for identical indications [9]. These MIA tests were a significant improvement for preoperative testing compared to single biomarker tests because of increased sensitivity (Table 2) [10]. Importantly, these tests are not true diagnostic tests, but rather triage or referral tests. When a woman is known to have an ovarian tumor that requires surgery, these tests are used to determine the likelihood of malignancy. A primary care provider can utilize the test to determine whether referral to a gynecologic oncologist is indicated. These tests have two critical requirements: (1) a mass has been confirmed on imaging, and (2) the ovarian tumor has already been determined to require surgery. Since the test itself is not used to determine whether or not surgery is necessary, it should result in minimal tangible harm. Nationwide, the majority of ovarian cancer surgeries are not initially performed by a gynecologic oncologist, so the hope is that the quality of patient care and cancer survival will improve over time as appropriate referrals are made. Provided that the two critical requirements are observed, this carefully considered strategy should prevent unnecessary surgery from a falsely positive biomarker test, an important consideration for the women, their doctors, and the FDA.

Multivariate index assays have continued to evolve. In 2016, the FDA cleared a new generation Ova1 test (Overa^®^) that essentially combines two MIA tests and maintains a high diagnostic sensitivity with improved specificity [12], Table 2. The individual markers are CA125-II, HE4, apolipoprotein A-1, follicle stimulating hormone, and transferrin. The preoperative indications are the same. Other panels will soon follow [13]. Naturally, there are always temptations to move a diagnostic test into a screening role, but without proper study, this is a premature and potentially harmful notion. Cancer screening and cancer diagnostics are vastly different challenges with regard to disease prevalence and endpoint objectives.

Ovarian biomarkers are not restricted to the blood. Ultrasound, like all imaging, is a biomarker of disease. Ultrasound has been widely studied in the United States and Europe as a screening tool and as a diagnostic adjunct. We are beginning to discover that ovarian ultrasound screening alone, or in combination with CA125, may have the potential to save lives [14,15]. Findings from the United Kingdom Collaborative Trial of Ovarian Cancer Screening (UKCTOCS) recently reported preliminary results of a shift to early stage disease and a reduction in cancer deaths on follow up to 14 years with multimodal ovarian cancer screening with serum CA125 interpreted using the Risk of Ovarian Cancer Algorithm (ROCA), transvaginal ultrasound, and clinical assessment. ROCA is an algorithm used to interpret longitudinal CA125 values for ovarian cancer screening. This story is far from over, but it is definitely premature to begin screening the general population off protocol. In fact, shortly following the UKCTOCS publication, the FDA, the American College of Obstetrics and Gynecology, and the Society of Gynecologic Oncology all made prompt safety statements announcing that ROCA is not an approved screening strategy and may trigger unnecessary surgical procedures.

How we combine biomarkers has a significant impact on their overall test performance. Tests can be combined in series or parallel. When combined in series (A, B and C, etc.), the statistical consequence is improved specificity at the expense of sensitivity. Conversely, tests combined in parallel (A or B or C, etc.) will result in improved sensitivity with a compromise in specificity. At the risk of oversimplification, the MIA tests are essentially combining individual biomarker tests in a parallel manner. Ova1 is a good example. Five biomarkers are applied in parallel in the same serum specimen with resultant high sensitivity (and high negative predictive value), making it an excellent triage test. If the test is low-risk, it is very unlikely to be malignant and the patient can have surgery without consulting a specialist. But the apparent drawback of this MIA strategy can be a modest specificity and ovarian tumors may have a high-risk test result even though cancer is not present. By requiring that a mass be confirmed on imaging prior to ordering Ova1, there is a mandate of sorts to combine an additional test (imaging) that localizes the problem to the ovary, improving both the sensitivity of finding an abnormality and the specificity that the problem arises from the ovary (though not that it is necessarily malignant).

**Figure 1:**
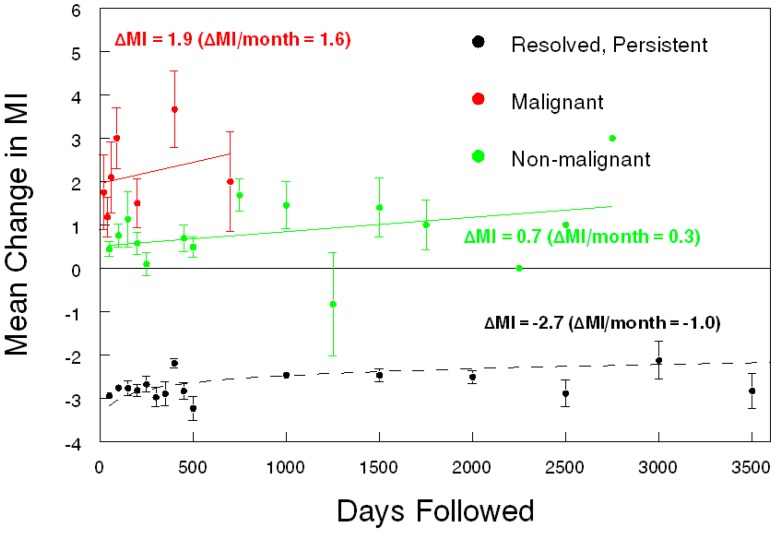
Results of serial ultrasound evaluation of ovarian tumors. MI, Morphology Index score, University of Kentucky (Lexington, KY, USA).

Today, serum biomarkers alone are not enough. In developed countries, there is no practical way to divorce serum biomarkers from ovarian imaging since ultrasound and CT scan are ubiquitous tests available to nearly every woman. Ultrasound is far less expensive than a CT scan or MRI, but ultrasound findings are limited mainly to the pelvis. An ultrasound-based morphology scoring system is an effective and objective way to identify ovarian tumors at high-risk for malignancy. The International Ovarian Tumor Analysis group (IOTA) has a multifaceted algorithm that has been systematically evaluated in Europe to high acclaim [16]. There have also been attempts to simplify the IOTA algorithm [17,18], and IOTA has yet to be evaluated in the United States. Other morphology-based indices have been proposed and validated in the U.S. and abroad [19,20,21]. Moreover, much like longitudinal CA125 (ROCA), serial ultrasound offers improved diagnostic results over a single evaluation (Figure 1) [22,23]. Serial ultrasonography is a sensible approach because each tumor is evaluated both on its changing complexity and its physiologic evolution. There can be clinical reasons not to perform serial evaluations on women with ovarian tumors. First, the presentation may be so concerning for malignancy that prompt surgery is best. Second, the woman may be symptomatic from the tumor so delayed intervention is problematic. Third, the patient may be traveling a great distance or have other personal reasons why a delay in treatment is not feasible. In the absence of these issues, a thoughtful re-evaluation is a valuable diagnostic option, and the data support this concept for serum CA125 in ovarian cancer screening (ROCA) and serial ultrasound with a quantifiable morphology index score in ovarian diagnostics (and maybe screening). The coup de gras, given our present diagnostic capability, would be a combination of serial MIA biomarkers with serial ultrasound. This data has yet to be published.

## 3. Yet-To-Come

Dickens was artful in his portrayal of Ebenezer Scrooge, allowing him to see his unflattering future through Marley’s ghost of Christmas yet-to-come. Of course, after his apparitional vision on Christmas Eve, Scrooge awoke transformed. And transformation is what we need for ovarian cancer diagnostics. It is certainly possible that new innovations will give rise to novel diagnostic insights, just as cancer therapy is trending toward targeted, molecular-based treatment. Although personalized cancer treatment is still far from the standard of care, it does raise the question, “Can we pursue a similar evolution in ovarian cancer diagnostics?” After 50 years, it is regrettable that we are still searching for effective approaches to early cancer diagnosis, but we are. As we transition our thinking and our oncology research to a molecular genetic model, we will recognize that this will unite malignancies in a different way, based on common molecular footprints rather than on an anatomic location or a given oncology specialty.

In the near term, we will see new types of serum cancer biomarkers that outperform our current protein-based markers in both selectivity and accuracy. Nucleic acids are showing promise as a new group of serum markers, including free DNA, mRNA, microRNAs, and circulating tumor DNA (ctDNA) [24,25]. A thoughtful combination of protein and nucleic acid markers may permit a comprehensive screening and diagnostic panel that captures all gynecologic malignancies in one blood test. In the future, an annual pelvic ultrasound and a comprehensive serum biomarker screening/diagnostic panel may replace the much maligned bimanual examination as part of the annual gynecologic examination. If abnormal, repeat testing will provide a personalized, serial database that will recalculate the likelihood of malignancy based on the objective change over time in tumor morphology and physiology. As the diagnosis and treatment of cancer changes, so too must clinical trial design to accommodate the new era of multiple biomarkers and targeted, personalized therapies [26].

Beyond the near future, germ-line cancer testing will be initiated at birth as part of newborn screening. Today, we often recommend genetic cancer testing following a malignant diagnosis, which is helpful for their future screening and for their relatives, but it is obviously a little late to prevent their own cancer. The power of knowing individual genetic risk at birth is that it may potentially modify behavior in those found to have a germ-line mutation, which comprise 5%–10% of cancers, and permit selective screening algorithms that are customized to personal cancer risk. And periodic genomic screening throughout one’s lifetime may help identify acquired mutations that predispose to specific cancers, heighten awareness, alter personal behavior, and dictate medical surveillance. The technology to sort, store and personalize this colossal amount of data is available today, a consequence of Moore’s law whereby computer processing speeds and power have roughly doubled every two years beginning in the 1960s. Cancer testing will quickly move beyond organ and specialty-specific screening. Whole body scans and universal cancer panels will screen and monitor all cancers, solid and hematogenous. An asymptomatic patient may not even need to see a physician if the annual evaluation is normal. A new medical field for screening and early diagnostics will emerge for physicians and epidemiologists, a field of study that is independent of patient gender, organ, or the subspecialties of today.

To get there, we must agree to work with industry innovators in medicine, technology and finance to develop and fund novel strategies for diagnosis and screening. We must encourage the national collaborative groups and the National Cancer Institute’s Clinical Trials Reporting Program to promote screening and diagnostic trials with as much vigor as the interventional treatment trials. Since the early detection of any cancer has the promise of shifting diagnosis to an earlier stage, cancer survival will improve. This approach could ultimately revolutionize how we provide care for our patients, and perhaps spare us yet another salvage chemotherapy trial for relapsed ovarian cancer.

So let us awake on a future Christmas morning with newfound clarity. Let us transform how we categorize ovarian cancer, how we identify ovarian cancer, how we treat ovarian cancer, and possibly how we screen for cancer in general. It did not take long for the Nuclear Age to change our worldview or for the Information Age to profoundly alter our daily lives; with any luck, it will not take long to revisit our approach to early diagnostics for ovarian cancer. If Ebenezer Scrooge can change his ways…

## Figures and Tables

**Table 1:** Common serum biomarkers for ovarian cancer, year of publication or Food and Drug Administration (FDA) clearance. CEA, carcinoembyonic antigen; CA125, cancer antigen 125; ROMA, Risk of Ovarian Malignancy Algorithm; HE4, human epididymis protein 4; Ova1 and Overa are proprietary multivariate index assays, Vermillion, Inc.

Biomarker	Year
CEA	1965
CA125	1981
HE4	2008
Ova1	2009
ROMA	2010
Overa	2016

**Table 2:** Test performance for detecting ovarian cancer of all histologic types.

Biomarker	Sensitivity	Specificity
CA125 *^,+,#^	76%	94%
Ova1 *	94%	54%
ROMA ^^^	89%	83%
Overa *	91%	69%

* Studied in same patient population; ^+^ CA125-II assay (second generation); ^#^ CA125 not FDA-approved for preoperative use; ^^^ Meta-analysis [11]
